# A Doleful Picture

**Published:** 1888-04

**Authors:** 


### A DOLEFUL PICTURE.

In the course of his recent Lent sermon Dr. Morgan Dix, rector of Trinity, discourses thus on the evils of New York fashionable society, which, if not overdrawn, should make every honest working girl feel proud of her superior station :

"Look how young girls are trained—in softness and luxury, with the one idea of making a figure in society and a brilliant marriage ; of making the most of their physical advantages, and alluring the other sex by the acts best adapted to that purpose. See them on the drive, through the troubled social sea ; at their lunch parties, with a dozen courses and half as many kinds of wines ; at the opera, immodestly attired ; at the ball, giving the whole night to dissipation ; at the summer haunts of fashion, without due oversight or sense of responsibility, treated with easy familiarity by careless men, and apparently without a vestige of an idea of what is due to a gentlewoman from a man. Listen to the low gossip among these young women ; to the broad speeches and unclean stories, by which they are prepared for the final surrender of the last ideas of propriety and of all faith in the honor and virtue of men.

Then pass on, and let us look at the woman as married—married, perhaps, for her money, or marrying some man for his money, without love and without respect ; married, but with no idea of living thereafter under bonds ; resolved to be more free and to enjoy life more ; eager for admiration, athirst for compliments and flattery, so that the husband early drops into a secondary position, and some other man, who does the madly devoted for the time, engrosses the larger share of her thoughts. Follow out this subject till you come to the divorce suit and the separation, and thence to the next, and now adulterous marriage, when those whom Christ and the Gospel forbid to marry so long as some one else liveth, snap their fingers at the attempted restriction, and commence a second partnership without fear and without remorse.

We all know that these are the commonest things of the day. We see men freely moving in high places whom no respectable woman should permit to cross her threshold ; notorious immorality condoned for the sake of great wealth ; grave social scandals, widely known and openly canvassed, though the actors are received with open hand and made welcome as before ; flirtations going on between persons, each of whom has plighted troth to some one else, and thus stands perjured before man and God ; men languishing after the wives of other men, and married men running after young girls and paying them attention, with the devil's look in the eyes and the devil's thoughts in the heart ; and women, young and old, permitting these demonstrations, agreeably entertained and flattered by them, and glad to find themselves still able to make conquests."

